# Analysis of plant cuticles and their interactions with agrochemical surfactants using a 3D printed diffusion chamber

**DOI:** 10.1186/s13007-023-00999-y

**Published:** 2023-04-01

**Authors:** Lakshmi Venkatesha Manyu Vittal, James Rookes, Ben Boyd, David Cahill

**Affiliations:** 1 Faculty of Science Engineering and Built Environment, School of Life and Environmental Sciences, Deakin University, Geelong Waurn Ponds Campus, Geelong, VIC 3216 Australia; 2 Department of Pharmacy, University of Copenhagen and Monash Institute of Pharmaceutical Sciences, Monash University, Parkville Campus, 381 Royal Parade, Parkville, VIC 3052 Australia

**Keywords:** Plant cuticle membrane, 3D printed labware, Diffusion analysis, Commercial surfactants

## Abstract

**Background:**

Decades of research is available on their effects of single component surfactant on active ingredient diffusion across plant cuticular membranes, but ingredient diffusion is rarely analysed in the presence of commercial surfactants. Also, diffusion studies require expensive or specialized apparatus the  fabrication of which often requires skilled labour and specialized facilities. In this research we have addressed both problems where the effects of four commercially available surfactants on a known tracer molecule were investigated using a 3D printed customized diffusion chamber.

**Results:**

As a proof-of-concept a customized 3D printed diffusion chamber was devised using two different thermoplastics and was successfully used in a range of diffusion tests . The effect of various solvents and surfactants on *S. lycopersicum* cuticular membrane indicated an increased rate of flux of tracer molecules across the membranes. This research has validated the application of 3D printing in diffusion sciences and demonstrated the flexibility and potential of this technique.

**Conclusions:**

Using a 3D printed diffusion apparatus, the effect of commercial surfactants on molecular diffusion through isolated plant membranes was studied. Further, we have included  here the steps involved in material selection, design, fabrication, and post processing procedures for successful recreation of the chamber. The customizability and rapid production process of the 3D printing demonstrates the power of additive manufacturing in the design and use of customizable labware.

**Supplementary Information:**

The online version contains supplementary material available at 10.1186/s13007-023-00999-y.

## Introduction

One of the widely used and environmentally sustainable way of delivering active ingredients (AI) in agrochemicals such as herbicides or nutrients to plants is through foliar sprays [1]. Efficient application of foliar sprays depends on several factors, such as formulation of sprays [2, 3], application methods [4, 5], reduced off target deposition [6], and environmental factors such as rainfall [7], high temperature, and low humidity [8, 9]. Hence the time of foliar application and the weather condition shortly after application are crucial for improving spray efficiency. Foliar sprays in general are a three-component system consisting of- (1) hydrophobic or hydrophilic active ingredients (herbicides, pesticides, nutrients) mixed with, (2) water (the universal solvent) and (3) chemical additives (adjuvants, surfactants). Upon reaching plant surfaces, foliar sprays containing water soluble-AI (nutrients, herbicides, pesticides), must cross a chemically tough, biologically inactive, hydrophobic, cuticular membrane complex (encompassing crystalline and amorphous waxes) covering the aerial plant surface.

Cuticular membranes form diverse physical and chemical barriers to a wide range of chemicals and biological organisms [10]. The diversity in the physical structure and chemical constituents of the cuticular membrane differ at different stages of plant growth [11, 12], due to environmental factors [13, 14] and differs between species [15, 16]. Diffusion of AI through the complex cuticular membrane (CM) is enhanced by mixing specific agrochemical additives called surfactants in the foliar spray mix. Surfactants, in general, are amphiphilic compounds with a polar head and non-polar tail. Surfactants enhance penetration by physicochemically interacting with crystalline and amorphous waxes and by disturbing the cuticular architecture and consequently being  adsorbed in the interfaces and by altering the surface or interfacial free energies of those surfaces [17].

Molecular penetration and the effect of several chemicals including ions, solvents, and surfactants on CM have been studied for over four decades [18]. Review of the literature into permeability studies through the plant cuticle indicated that the majority of these studies often included the interaction of individual chemicals [19] or individual chemical with single surfactants [20] with cuticular membranes. On the other hand, commercial surfactants like the ones that are used in this research are often combination of two or more chemicals, a typical end user product that has not been investigated. Recent market data on agrochemicals indicate that there are nearly one thousand commercial surfactants actively used in the world with over 300 surfactants just used in Australia [21], but very little information is available around the permeability analysis of these commercial surfactants [22]. In addition, cuticular diversity, diversity in surfactant formulations significantly adds complexity to molecular diffusion studies. Understanding the surfactant- cuticle interaction is vital, as excess usage of foliar chemicals can result in chemical imbalance in the target plant system, environmental damage due to chemical runoffs, and an increase in the cost of usage and resources. In this research work more focus was  put towards the development of a diffusion apparatus using a user-friendly approach and using the developed chamber for permeability studies and  less focus on discussing the biomechanics of cuticular membrane.

Fifty years of research into molecular diffusion across plant membranes has often resulted in custom design and fabrication of diffusion chambers [18]. Expensive alternatives like Franz cells (a borosilicate diffusion apparatus generally used in animal skin permeability studies) has also been used in AI diffusion studies through leaves [23]. The materials used for fabrication have conventionally been either glass or stainless steel [24–26] and recently Perspex [27]. The problem in using glass or stainless steel is that they require specialized facilities and expertise to fabricate complex designs. Further, design modifications to the prototype can require manufacturing a new modified prototype resulting in excess time and cost. As an alternative, in this research we customized and fabricated a diffusion chamber using readily available, malleable, low-cost plastic through additive manufacturing.

3D printing or additive manufacturing allows the fabrication of geometrically complex physical objects in a layer-by-layer fashion, with a click of a button. Since its introduction in the 1980’s the application of this technology has diversified [28, 29]. In biological sciences, 3D printing has been used to print customized labware [30], microfluidic devices for DNA assembly studies [31], reaction ware [32], diffusion chamber for bacterial cells [33] and spare parts [34]. The methods and materials available for 3D printing are diverse and do not impose any limitation on the complexity of the design. The most readily available and cost-effective method of fabrication is fused deposition modelling (FDM). FDM printers work on thermoplastic extrusion, where a plastic filament extruded through a heated nozzle is deposited, using a computer aided program, in vertical-horizontal directions as layers of molten plastic.

The primary aim of this research was to understand the effect of four readily available commercial surfactants manufactured and acquired from Victorian Chemical company^®^ (Coolaroo, Australia) and some readily available solvents on a hydrophilic tracer molecule permeability immediately after application (in the first 6 h) through enzymatically isolated astomatous tomato fruit cuticular membranes. The diffusion study was carried out  in a custom designed and 3D printed diffusion apparatus, as a proof of concept, which is the secondary aim. The rate of exchange of molecules between the donor and receiver chambers that occurred due to concentration differences was determined  by calculating the flux. In addition, the physiological effects of surfactants and solvents on isolated cuticular membranes was investigated using scanning electron microscopy.

## Materials and methods

### Enzyme isolation of fruit cuticular membrane

Cuticular membranes of *Solanum lycopersicum* (procured from a local supermarket) fully ripe fruits were enzymatically isolated using a standardized method [35]. Briefly, fresh fruits were washed with sterile distilled water, de-crowned and were cut into four quadrants using a sterile scalpel ~ 1 cm from the crown. The mesocarp and underlying tissue with seeds were discarded from each cut quadrant leaving a thin layer (~ 0.5 cm) of fruit epidermis and skin, which were further cut to 5 cm × 5 cm sections. Cut sections were floated in a sterile glass tray with cuticle enzyme isolation solution incubated at 35 °C for ~ 48 h, and gently agitated every 12 h with a micropipette. The enzyme isolation solution consisted of 50 mM sodium acetate buffer (31 °C) prepared by mixing 2.44 g sodium acetate trihydrate (M. wt. 136.08 g/mol), 4.68 mL of glacial acetic acid (17.485 M), and 400 mL of autoclaved distilled water; adjusted to pH 4 using 1 M hydrochloric acid. To this buffer the isolation enzyme was added: a concoction of 8 mL of pectinase (10 U mL^−1^, Sigma Aldrich®, Australia), 0.4 mg of cellulase (1.3 units/mg, Sigma Aldrich®, Australia) and 26 mg sodium azide was added as an antibiotic. Partially isolated CM were subjected to acid and alkaline washes to completely remove epidermal over hangings as described by L Schreiber and J Schonherr [18]. Briefly, partially isolated CM were floated in 1 M HCL for 48 h, and then filtered under vacuum using a Buchner funnel and following wash with deionized water. Following this, CM were subject to borax buffer (pH ~ 9.0) wash for 24 h and further washed with deionized water three times. Isolated CM were dried in a gentle stream of nitrogen and stored in Teflon coated cooking pan set at 25 °C covered with baking paper (does not stick to the isolated CM) and small glass petri dishes as weights (to prevent curling of the isolated CM) were placed over this set up to hold CM flat overnight for further drying. All Isolated CM were visually inspected for holes and tears under a microscope and only intact CM were selected for permeability studies.

### Optical and electron microscopy

To understand the architecture of the isolated cuticular membrane and the effect of solvent, and surfactant on CM, fresh and treated CM were cut to small squares of size 1 cm × 1 cm and were carefully placed on SEM aluminium stud with a double-sided conductive carbon tape stuck to its surface. Around 80% of the tape surface was covered with CM (the adaxial side visible) and the CM were rolled up perpendicular to the surface of the stud. This was to visualize the underside (abaxial side) of CM. Studs loaded with CM were coated with 5 nm thick gold particles using the Leica EM ACE 600 carbon and e-beam coater (Leica Microsystems^®^, USA). Gold coated samples were visually inspected at 10 keV and at high vacuum (~ 2 × 10^−3^ Pa) using the electron microscope Jeol® In-touch™ JSM- IT 300 (Jeol^®^, USA). Like the fresh CM, the solvent and surfactant treated CM were coated with gold using the sputterer and were visually inspected at 10 keV and at high vacuum using the table-top scanning electron microscope Jeol Neoscope™ JCM-5000 (Jeol^®^, USA). For every treatment (fresh, solvent and surfactant) 20 CM were measured for thickness using the onboard measuring tool. Each CM was randomly measured three times across the CM and the averages were used for estimating the thickness of the CM. The architecture of fresh cuticular membranes were also visually observed under light microscope Zeiss Axioscope 2 plus (Zeiss, Germany). Investigation under the light microscope was carried out by trimming down air dried isolated cuticular membranes (2 cm × 2 cm) and placing them on glass slides with the adaxial side facing up. The isolated CM were directly observed at 2.5x, 40 × and 63 × objective magnification.

### Chamber fabrication

#### Designing the diffusion chamber using 2D and 3D software

**Fig. 1:**
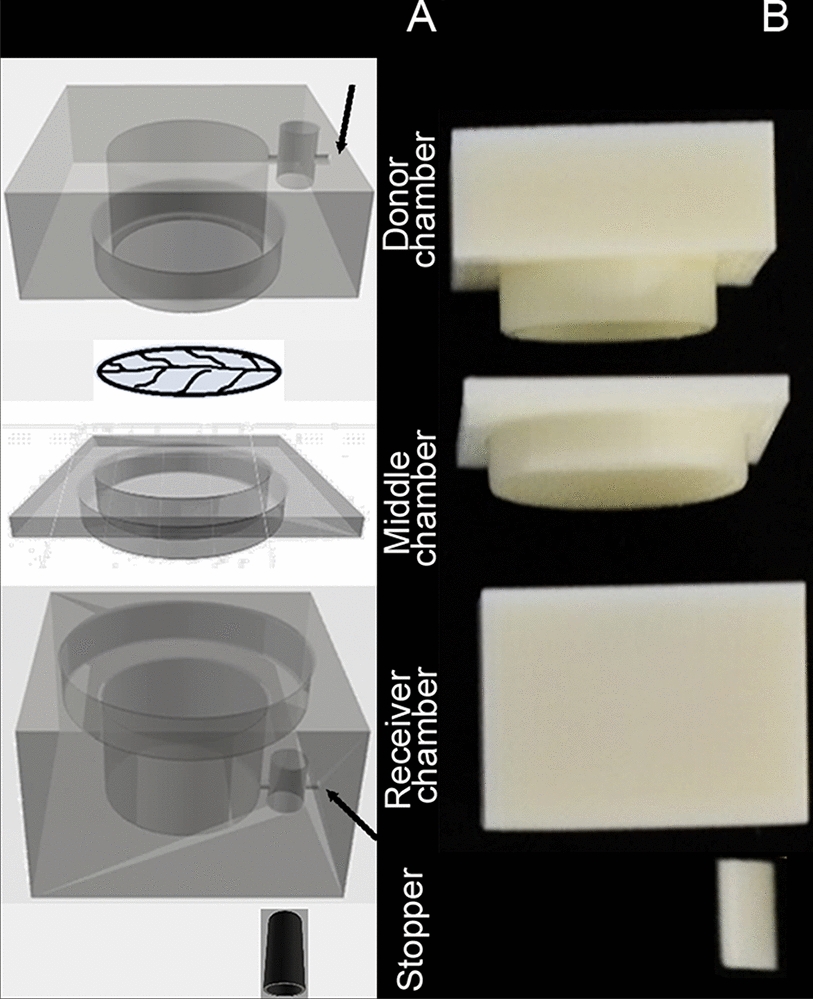
Axonometric digital view and side view of the printed chamber. **A** represents the different parts of the diffusion chamber in an axonometric view and **B** represents a printed 3D chamber. In image A the four parts of the chamber show the inside contours of the different parts of the chamber. Sampling port canals (black arrow) can be seen as a thin horizontal line towards in the larger cylindrical section in the donor and receiver chamber from the outside. The leaf like diagram between the donor and middle chamber is an artistic representation of cuticular membrane. Image B represents a completely printed diffusion chamber printed with ABS (ivory) P430 thermoplastic. Image not to scale.

The 3D printed diffusion chamber designed for this experiment was based on a functioning stainless steel diffusion chamber kindly provided by Professor Lukas Schreiber, Department of Ecophysiology, Institute of Cellular and Molecular Botany, University of Bonn, Germany. The diffusion chamber designed for printing had five components: (1) top chamber/donor chamber, (2) middle chamber/the adaptor, (3) bottom chamber/receiver chamber, and (4 & 5) plugs for sampling port. Once a hand-drawn model design was drafted, technical drawings of the different parts of the diffusion chambers were made using Autodesk^®^ AutoCAD LT 2018^®^ edition. The final measurements of the diffusion chamber are represented as 2D technical drawings (10:1 scale) in the Additional file 1: (Figure S1). Measurements from 2D CAD designs were used to create a 3D solid form using Autodesk^®^ Fusion 360™ version 2.0.3034 and Autodesk^®^ Print studio™ version 1.6.5 to edit printer parameters for printing. Fusion 360™ and Print studio™ programs were available to download from Adobe. An axonometric view of the final design of the chamber is shown in Fig. 1 and the various steps involved in forming the final.STL file of the top chamber is described in the series of screen-shot images in the Additional file 2: (Figure S2.1–S2.7). A digital reconstruction of different parts of the chamber can be viewed using the links provided in the Additional file 3

#### Material selection

Thermoplastics were selected for chamber fabrication based on chemical, and auto fluorescence compatibility. Small 5 mm × 5 mm × 2 mm 3D printed sections of PETG (polyethylene terephthalate glycol copolymer), P430 ABS, ABS M30 (Acrylonitrile butadiene styrene), nylon and vero magenta (Stratasys®, Israel) were used for compatibility testing. For chemical compatibility testing printed samples were weighed before and after solvent treatments (at 1 h and 24 h post treatments). The test included the following five solvents at neat concentrations- distilled Milli-Q™ water, acetone (analytical reagent), chloroform (analytical reagent), 2- propanol (analytical reagent), and acetonitrile (HPLC grade); all solvents purchased from Thermofisher Scientific^®^, Australia. A similar test was conducted against neat concentrations of four surfactants esterified vegetable oil (EVO), fatty acid ethoxylate (FAE), organosilicone (OS), and alcohol alkoxylate (AA) supplied by Victorian Chemical Company^®^, Australia.

#### Printing and processing parameters

The .STL files were used to print the chamber at the Centre for Advanced Design in Engineering Training (CADET), Deakin University. Preliminary tests were carried out using a range of printing substrates; ABS (Acrylonitrile butadiene styrene) in P430 (ivory) and M30 (white) and PETG (Poly-ethylene terephthalate glycol) (black), nylon (translucent), and Vero magenta (proprietary information) (bright pink). These FDM materials were printed using a range of printers uPrint^®^, Fortus 450MC^®^, FlashForge^®^, and Object500^®^ (Stratasys, Israel). The parameters used in printing are presented in Table 1. The materials were printed between 50 and 90% density in the infill region.

**Table 1:** Slice and print information, and printing parameters used to 3D print various FDM materials

**Process**	**Parameter**	Printers				
		Zortrax M200^®^	uPrint^®^	Fortus 450MC^®^	Flashforge^®^	Object500^®^
	Filament supplier	Stratasys	Stratasys	Stratasys	Stratasys	Stratasys
	Material	PETG	P430 ABS	ABS M30	Nylon	Vero Magenta
	L_T_ (mm*)*	0.09–0.39	0.254	0.127/0.178/0.254/0.330	0.2–0.4	0.02–0.04
**Slicing**	R (z/xy) (mm)	0.13/0.26	0.13/0.26	0.13/0.26	0.13/0.26	0.01/0.03
	Infill solidity ^a^	1	1	1	1	1
	Infill pattern	Line	Line	Line	Line	Line
	Shell number ^b^	3	3	2,3	3	3
**Printing**	Speed (mm s^−1^)	25	25	25	25	25
	P_I_ (mm s^−1^)	40	40	40	40	40
	Extruder temp. (°C)	200	240	240	255	250
	Print bed temp. (°C)	60	110	110	100	75
	Extruder fan	Yes	No	No	Yes	Yes
	Brim	No	No	No	No	No

^**a**^ Ratio of the filament to air in the interior part

^b^ Number of adjacent threads of filament that outline all structures

*L*_*T*_ layer thickness, *R* resolution, *P*_*I*_ perimeter infill

Inside of the printed chambers were processed through mechanical abrasion or manually using sandpaper. Printed chambers were malleable hence the inside of the chamber was smoothened using Dremel with rotary sanding head grit 60 (Bosch^©^, UK) and sandpapers at 1000 and 2000 grit (Raptor supplies™, UK). An orifice was drilled into the middle chambers with a 1 cm diameter drill bit. Leakage tests were conducted using 0.05% w/v toluidine blue to identify gaps in the printed chambers. Leakages were addressed by two methods in already printed chambers the inside of the chambers were coated with a thin coat of XTC-3D^®^ (Smooth-on^®^, USA) and air dried for 24-48 h. For new chambers leakages were stopped by simply increasing the infill density from 50 to 100%.

### Diffusion experiments

#### Permeability through non biological membrane

Molecular permeability of a tracer (fluorescein sodium salt) was calculated as flux through a series of materials. A 50 µM working stock solution was prepared from 500 µM stock solution by diluting 5 mL of the stock in 45 mL of water. The non-biological material tested here were printed plate (ABS and PETG) (middle chamber 2000 µm thick), aluminium foil: ⁓20 µm thick (Confoil^®^, Australia), Selleys^®^ silicone sealant: ⁓460 µm thick (Selleys^®^, Australia), and Spectra Por1 dialysis membrane: ⁓40 µm thick and MWCO (molecular weight cut-off) of 6–8000 Da, (Repligen Corporation^®^, USA). To activate the dialysis tube, 2 cm × 2 cm cut sections of the tube soaked in distilled water for 30 min, followed by a 10 min soak in autoclave sterile Milli Q™ water and then washed in 1% w/v sterile EDTA solution. EDTA wash solution was prepared by dissolving 1 g of EDTA disodium dihydrate in 99 mL Milli Q™ water. Following EDTA wash, the dialysis membrane was washed three times in sterile distilled water and floated in fresh sterile Milli Q™ water prior use.

The diffusion experiment was carried out by injecting 2.8 mL of the tracer working solution through sampling port in the donor chamber once, collecting 1 mL of sample in 1.5 mL Eppendorf^®^ Safe-Lock microcentrifuge tube (Sigma-Aldrich^®^, Australia), and replacing with 1 mL of deionised water from receiver chamber at different time points. The middle chamber was loaded with different materials and the tracer concentration was calculated at different time points using Varioskan™ Lux multimode plate reader (Thermofisher Scientific^®^, USA), an analytical spectrophotometer. Foil, dialysis membrane, and isolated CMs were encased between flat rubber O-rings and middle chamber with silicone sealant. The loaded diffusion chamber sealed with silicone sealant was placed flat (on its side) on a flatbed rocking incubator set at 25 °C at 2 rpm. All liquids used maintained at 25 °C in a water bath before injecting into the diffusion chamber. Frequency of sampling was at 24 h for 5 days for printed plates, silicone layer, and foil as we expected little to minimal diffusion. Sampling frequency was at 60 min for 24 h when dialysis membrane was used in the study. All experiments were repeated 10 times.

#### Permeability through biological membrane- fresh, solvent and surfactant treated CM.

Like the diffusion chamber set up with semi permeable membrane, enzyme isolated air-dried fresh CM segments (3 cm × 3 cm) were encased between rubber washer (2 cm diameter, with 1 cm diameter central orifice) (Zenith rubber^©^, Australia) and middle chamber using silicone sealant; with physiological outer side of the CM placed facing the donor chamber. For CM subjected to solvent treatment, enzyme isolated air-dried CM segments (3 cm × 3 cm) were floated in various 50 mL centrifuge tubes (Eppendorf^®^, Germany) containing neat concentration of chloroform, acetone, ethanol, acetonitrile, and 2-propanol (100% v/v, Ajax finechem^®^, Australia) for 24 h. Solvent treated CM were air dried for 24 h on Teflon pans and then loaded in the diffusion chamber as described for untreated CM. In case of surfactant treatments, the CM were loaded in the middle chamber and then 20 µL of neat concentration of esterified vegetable oil (EVO) commercial name Hasten^®^, fatty acid ethoxylate (FAE) commercial name Deluge^®^, alcohol alkoxylate (AA) commercial name Surewet^®^ and organosilicone (OS) commercial name Sprinta^®^ surfactants (Victorian Chemical Company^®^, Australia) were deposited and air dried for 24 h. The segments were then loaded in the diffusion chamber for permeability analysis. The diffusion of tracer through fresh, solvent and surfactant treated CM were conducted in the same manner as diffusion studies through non biological membrane; with sampling frequency at 60 min, total experiment duration at 24 h, and 20 repeats per experiment.

#### Effect of surfactants on tracer diffusion through isolated CM

The effect of surfactants at the industry recommended application concentrations of 0.01% v/v and 0.1% v/v was investigated, along with a higher concentration of 1% v/v. The recommended concentrations of the surfactants were added in the donor chamber along with 2.8 mL of water with 50 µM tracer once and regularly topping up with distilled water. Sampling frequency from the receiver chamber was at 30 min intervals for 6 h. Sampling frequency was increased to 60 min for 1% v/v surfactant concentrations and the experiment extended to 24 h as the fruit CM is known to be reasonably water permeable [18]. The experiments were repeated 20 times.

#### UV spectroscopy

Collected samples were analysed for tracer concentration under fluorescence spectroscopy using the Varioskan LUX™ multimode microplate reader (Thermofisher Scientific^®^, USA). From the samples withdrawn from the receiver, 100 µL was eluted in a 96 well blue coloured round bottomed well plate (Thermofisher Scientific®, USA). This was then observed under the excitation (λ_ex_ = 460 nm) and emission (λ_em_ = 515 nm) wavelengths, and the absorbance values were recorded. The absorbance values were substituted in the equation of the line obtained from the calibration curves and the concentration of the tracer was estimated. An eight-point calibration curve was generated from which the total concentration of tracer in water was calculated.

## Results

### The architecture of isolated cuticular membranes

**Fig. 2:**
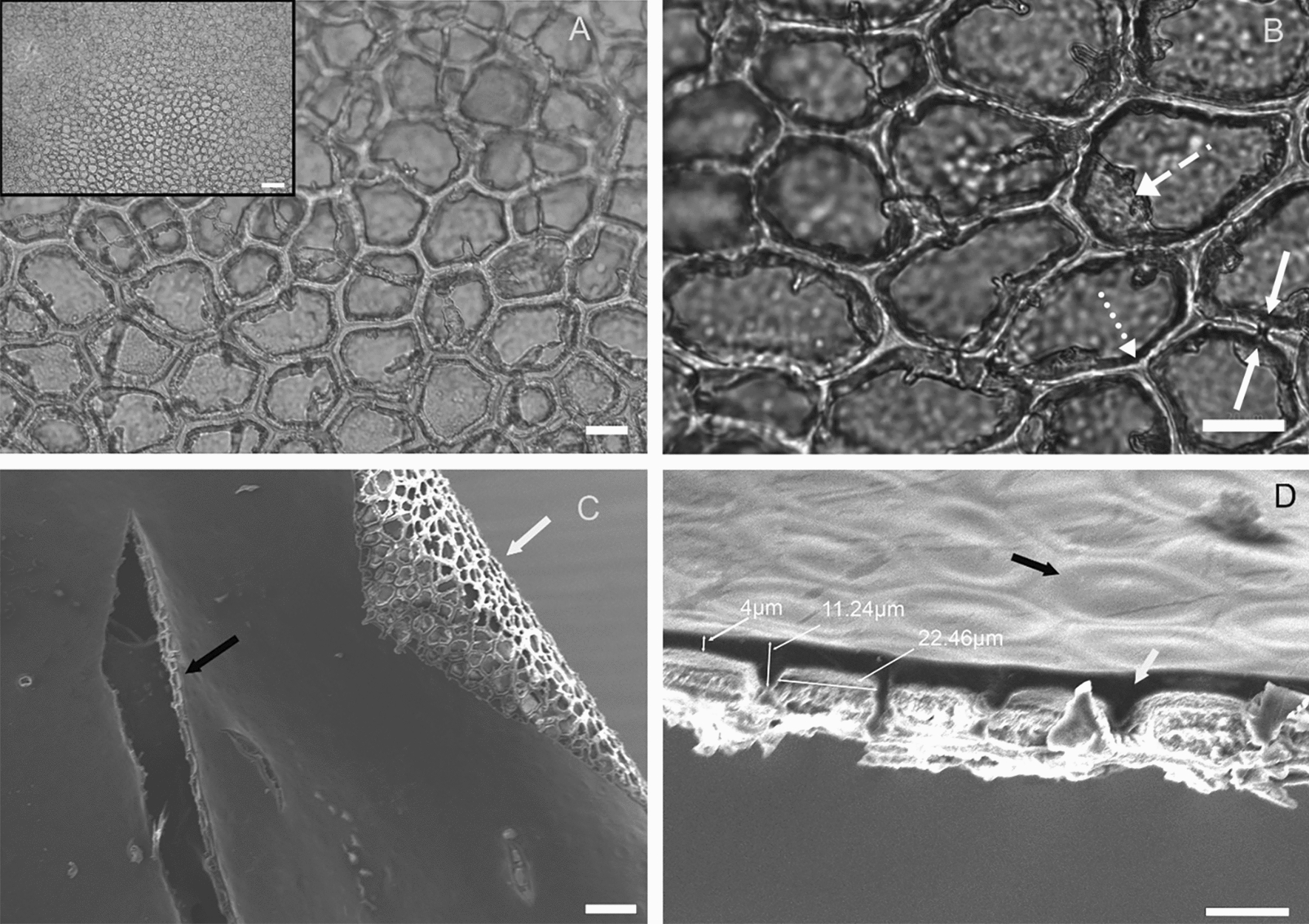
The architecture of the enzymatically isolated *S. lycopersicum* cuticular membrane. Greyscale image **A** and **B** represents the top view of enzymatically isolated fruit CM under light microscopy and **C** and **D** represents top, bottom, and lateral view of fruit CM images obtained using an SEM. Images A, inset, and B represents images of fruit cuticle in increasing magnification at 2.5x, 40x, and 63× objective magnification. Images A and inset shows the transparent epicuticular wax and many irregular polygonal empty epidermal cell pockets lined with thick anticlinal walls. Solid white arrows in image B shows gaps- cuticular canals. Dashed white arrow shows anticlinal walls overhangs and dotted arrows shows anticlinal regions. Image C shows physiological outside (top) and inside (bottom) of fruit cuticle. Black arrow indicates a mechanical tear during isolation and white arrow indicates epidermal cell pockets. Image D shows the transverse section of the fruit cuticle showing epicuticular layer light grey in colour with hexagonal patterns (black arrow) and occasional trichome. The white arrow indicates darker anticlinal region, and three measurements indicate thickness of CM at different sections and width of an anticlinal region. Scale bar in µm for images A, inset, B, C, and D- 28, 500, 20, 100, and 20 respectively.

Enzymatically isolated and air-dried (24 h) *S. lycopersicum* cuticular membranes were thin, pale orange in colour with a shiny outside (facing environment) and physiological (underlying fruit layers) matte inside. Under the light microscope at 25 × magnification the fruit CM appeared greenish-orange colour, displayed a transparent amorphous layer, and darker epidermal cell walls with clear centre (inset, Fig. 2A). At higher magnifications (100x, 200x, 400× and 630x) the darker spots showed irregular shaped-oval structures which were identified as epidermal cell pockets (Fig. 2A). Epidermal cell pockets were separated to the adjacent pocket by cuticular membrane ⁓1.3 µm thick called anticlinal region and this anticlinal region expanded to ~ 4.2 µm thick when three pockets intersected (Fig. 2B). Canal-like gaps (thickness ~  < 1 µm) were visible connecting neighbouring pockets in perpendicular direction to anticlinal walls (solid white arrow Fig. 2B), not visible through electron microscopy. Images collected through electron microscopy revealed bumps on the physiological outer side of the fruit CM. Other structures like trichomes, orifices, and mechanical tears (Fig. 2C and D) were also observed. The underside of the fruit cuticles showed regularly spaced voids- “epidermal cell pockets” separated by anticlinal walls of the cuticular membrane (Fig. 2C and D). Cross section views of the anticlinal regions revealed the thickness to be ~ 6.2 µm tapering towards physiological inside of the fruit (Fig. 2 D).

### Physical effect of solvents and surfactants on isolated cuticular membranes

**Fig. 3:**
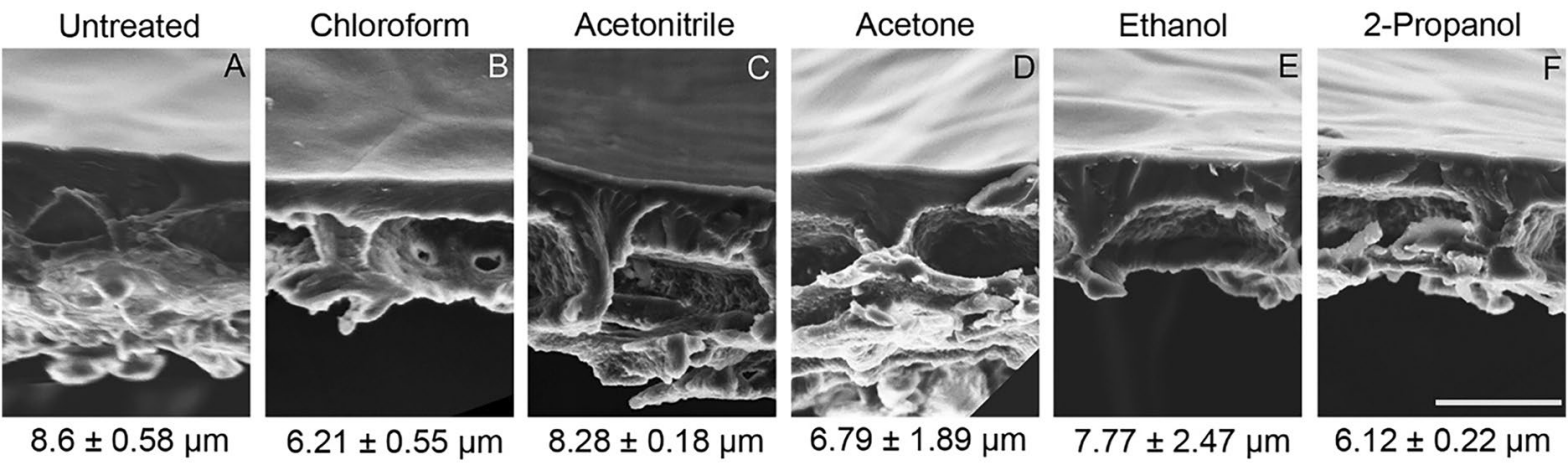
SEM images of transverse sections of fresh and solvent-treated isolated *S. lycopersicum* cuticular membranes. The figure represents the physical structure of the transverse section of solvent untreated and treated CM visualized under scanning electron microscopy, with respective average thickness and standard deviation measured. **A** Untreated isolated fruit CM showing thick CM layer with average thickness around ~ 8.6 µm. **B** Dewaxed tomato CM treated with chloroform with average thickness of ~ 6.21 µm and epidermal cell pockets. **C** Acetonitrile treated CM with average thickness around ~ 8.2 µm. **D** CM treated with Acetone showing average thickness of ~ 6.79 µm. **E** Ethanol treated CM with average thickness of ~ 7.7 µm. **F** CM treated with 2- propanol showing average thickness of ~ 6.21 µm. Scale bar 20 μm.

Electron micrograph of fresh, solvent and surfactant treated cuticular membranes showed differences in thickness and appearances. The outermost cuticular layers were dewaxed upon chloroform treatment only. Epidermal cell pockets and anticlinal pegs did not show any structural changes upon all solvent treatments. Fresh cuticular membranes were ~ 28% thicker than dewaxed CM (Fig. 3A, B). Sections of dewaxed CM showed a transparent crusty layer < 0.5 µm in thickness peeling from rest of CM. Mechanical tears and fissures that occurred during the enzymatic separation were most apparent after chloroform treatment. Colour of the CM became lighter upon 2-propanol treatment, and this colour change was not seen with acetonitrile, acetone, ethanol, and chloroform. The thickness of the CM treated with acetone and propanol reduced by ~ 21% and ~ 29% respectively (Fig. 3D and F). The CM were stable in acetonitrile and ethanol (Fig. 3C and E).

**Fig. 4:**
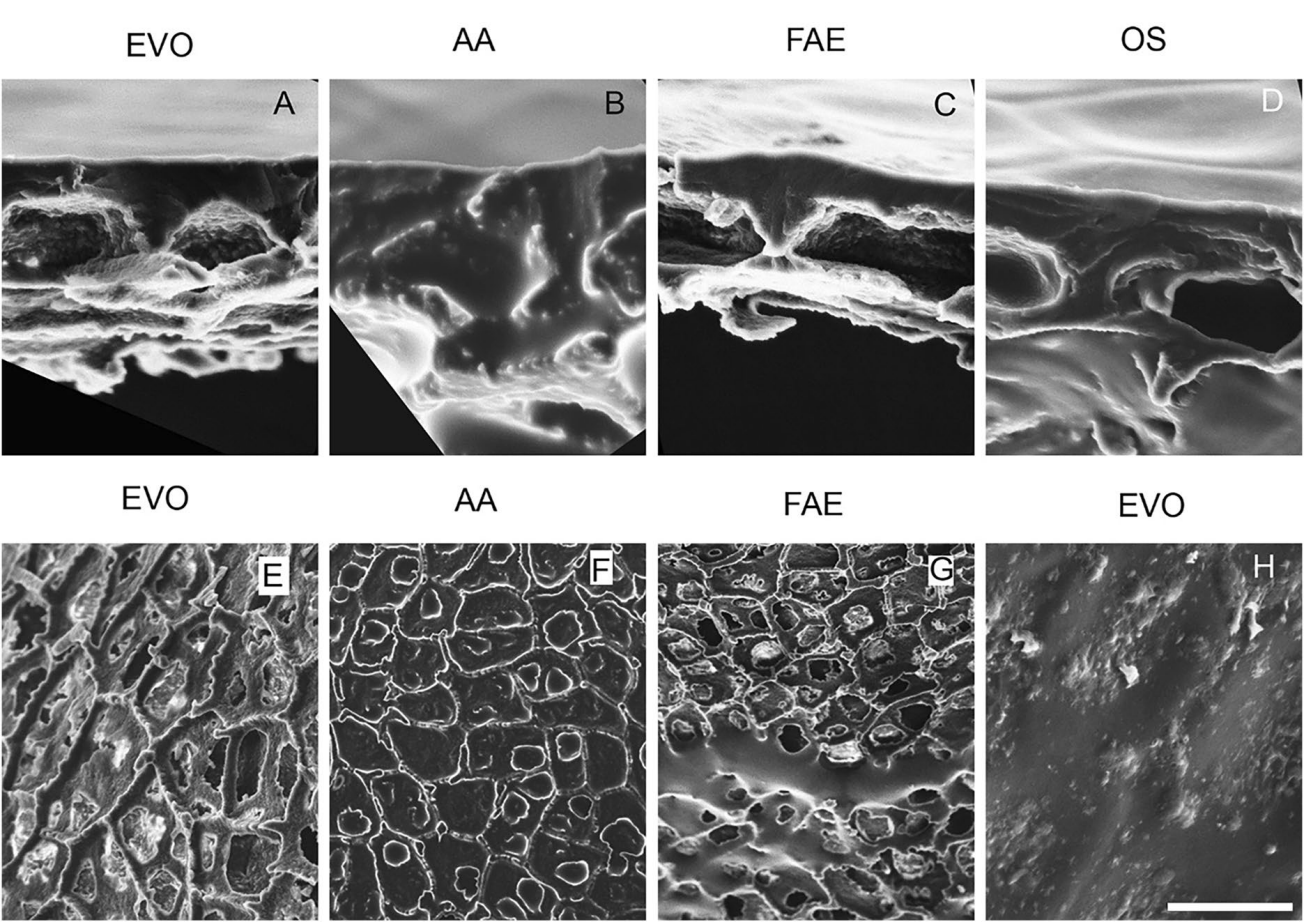
SEM of surfactant treated isolated *S. lycopersicum* cuticular membranes. **A**–**D** transverse view of enzyme isolated CM treated with esterified vegetable oil (EVO), alcohol alkoxylate (AA), fatty acid ethoxylates (FAE) and organosilicone surfactant (OS). Surfactant deposited on the physiological outside 24 h prior to the experiment were found on the physiological inside of the CM. The deposits of surfactants AA and OS were clearly visible in the epidermal cell pockets. **E**–**G** represents physiological inside and **H** represents physiological outside of surfactant treated fruit cuticular membranes. With respect to EVO and AA the deposits filled epidermal cell pockets and looked dried up. FAE and OS deposits also filled the epidermal cell pockets but also remained as liquid as seen in image **G**. Occasional pitting was observed on the physiological outside of the CM treated with EVO as shown in image **H**. Scale bar 20 μm.

CM treated with neat concentrations of surfactant deposits did not show reduction in cuticle thickness on the physiological outside. Instead, evidence of thickening was found on the underside side (pocket regions) as shown with white arrows in Fig. 4B–G. FAE and EVO treated CM showed occasional pitting (Fig. 4H). Certain sections of the CM did not show altered epidermal cell pockets (Fig. 4A and C). The epidermal cell pockets were clearly less hollow in CM treated with surfactants and evidence of this were seen in Fig. 4E–G indicating surfactant interaction with CM.

### Material selection, chamber design and fabrication of diffusion chamber

#### Design

The permeability chambers were initially digitally designed in a 2D format with a 10:1 scale using AutoCAD^©^ (Autodesk^®^, USA) Additional file 1. The 2D drawings were redesigned to a 3D form using Fusion 360^©^ (Autodesk^®^, USA). All models can be viewed in 3D using the following links in the Additional file 3. The various steps involved in forming the final.STL file of the top chamber is mentioned in the series of screen-shot images in the Additional file 2 for reference.

#### Material selection

Chemical stability was tested on five thermoplastics, to ascertain the stability of materials against solvents and surfactants used in this research. Chemical stability was assessed using visual inspection and change in weight. An in-depth overview of results is provided as a chart in the Additional file 3: (Figure S3). Briefly, the thermoplastics were treated against water, chloroform, acetone, acetonitrile, methanol, and 2-propanol. None of the thermoplastics reacted to water, whilst both the ABS- P430 (ivory) and M30 (white) were highly reactive and were completely dissolving in chloroform, acetone, acetonitrile. PETG dissolved in chloroform and showed a minor reaction to acetone and acetonitrile. ABS and PETG did not show any reaction to the other three solvents (40% acetonitrile, 2-propanol, and methanol). Vero magenta showed a severe reaction to chloroform and gained weight with all the solvents. Nylon was the most stable material showing visible reaction to the solvents. All the materials showed no reactivity to surfactants.

#### Fabrication

**Fig. 5:**
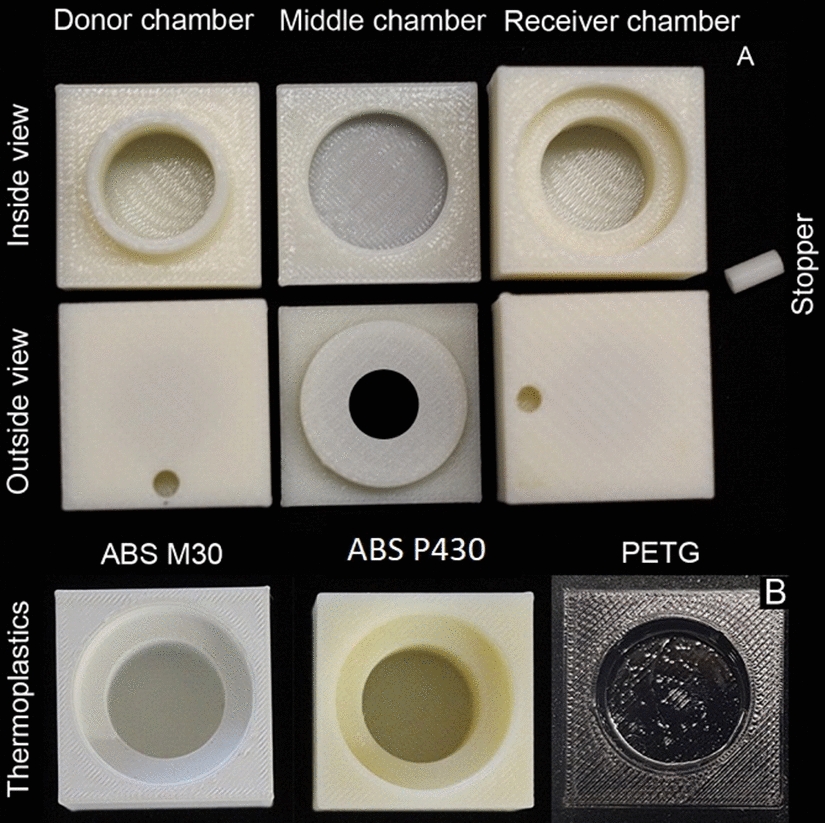
Photographs of 3D printed diffusion chambers. **A** is a photo representation of different parts of a diffusion chamber printed using ABS P430 thermoplastic. The top row indicates the inside view of the chamber, showing the cylindrical interior with interlocking contours. Donor chamber fits in the middle chamber (without a hole) and this setup fits into the receiver chamber. The outside view shows rectangular blocks with smaller cylindrical shafts for stoppers, middle chamber printed chamber with a central orifice, and cylindrical stopper. The isolated cuticular membranes are housed in the middle chamber molecular exchange between and donor and receiver chamber occur through this hole. **B** is a photo collection of the three thermoplastics used in this research with and without surface coating; ABS M30 (white) uncoated, ABS P430 (ivory) uncoated, and PETG (black) coated with XTC 3D appearing as the shiny centre. Image not to scale.

Thermoplastics ABS P430, ABS M30, and PETG were selected based on chemical stability for printing diffusion chamber. Printed chambers are shown in the Fig. 5. On visual inspection, some of the initially printed chambers showed gaps between the deposited layers. This was rectified by increasing the infill density from 50 to 100%. An orifice was drilled into the middle chambers with a 1 cm diameter drill bit. The inner walls of the printed chambers were smoothened either mechanically or manually using sandpaper. This was not necessary for chambers printed with PETG thermoplastics as the printed material had an exceptionally smooth finish. Further, the printed chambers were tested for leaks using a 0.1% toluidine stain (w/v). Chambers with gaps in the walls were sealed by applying a thin coat of XTC 3D® and was air-dried for 24–48 h. To maintain consistency XTC 3D® was used on all ABS printed chambers. This coat was not necessary for chambers printed using PETG thermoplastic as they did not show any gaps or had leakages.

### Permeability of tracer

The flux of the fluorescein sodium salt (tracer) through different materials were calculated by generating an eight-point calibration curve. Flux of the tracer was calculated at 24 h time point. As expected, the flux of the tracer through semi permeable membrane dialysis Spectra Por1 in 24 h was over 600 times higher when compared with tracer diffusion through foil and silicone. The tracer diffused through isolated tomato fruit cuticular membranes almost immediately. Chloroform treated, (i.e., dewaxed) cuticular membrane showed the highest amount of tracer permeability and showed a significant difference compared to fresh or cuticular membranes treated with other solvents. There was no significant difference in the amount of tracer diffused between cuticular membranes treated with ethanol, acetonitrile, acetone, and fresh cuticles. On the other hand, significant amount of tracer diffused through 2- propanol treated CM when compared with CM treated with other solvents.

The flux of the tracer through non biological material, fresh and solvent treated CM are listed in the Table 2 and amount diffused in Additional file 5: Table S2. Chloroform treated cuticular waxes showed nearly eight times more molecular permeability of the tracer compared to that of tracer diffusion through fresh CM. Tracer permeability was at the highest for dewaxed cuticular membranes compared to that of fresh and surfactant treated cuticular membranes. Fatty acid ethoxylate, esterified vegetable oil and alcohol alkoxylate showed similar tracer flux but certainly an increased flux when compared to flux of tracer through fresh CM. Organosilicone surfactant treated cuticular membranes show reduced tracer flux in comparison with CM treated with other surfactants. These results suggest that organosilicone surfactant treated CM showed the lowest change in permeability of tracer compared to that of the CM treated with other surfactants. The flux of the tracer through surfactant treated is tabulated in the Table 2.

**Table 2:** Flux of tracer through treated and untreated materials

Material	**Treatment**	Flux mol m^−2^ s^−1^ ± SD
*Non biological membrane*		
Foil	Nil	6.8 × 10^–11^ ± 1.71 × 10^–11^
Silicone	Nil	6.57 × 10^–11^ ± 2.3 × 10^–11^
Membrane	Nil	8.56 × 10^–5^ ± 0.11 × 10^–5^
*Biological membrane*		
CM	Fresh	1.4 × 10^–5^ ± 0.13 × 10^–5^
*Solvents*		
CM	Acetonitrile	4.4 × 10^–5^ ± 0.02 × 10^–5^
CM	Ethanol	5 × 10^–5^ ± 0.16 × 10^–5^
CM	Acetone	5.3 × 10^–5^ ± 0.01 × 10^–5^
CM	2- Propanol	7 × 10^–5^ ± 0.14 × 10^–5^
CM	Chloroform	8.2 × 10^–5^ ± 2.19 × 10^–5^
*Surfactants*		
CM	OS	4.4 × 10^–5^ ± 0.15 × 10^–5^
CM	EVO	6.05 × 10^–5^ ± 1.5 × 10^–5^
CM	FAE	6.2 × 10^–5^ ± 0.08 × 10^–5^
CM	AA	6.8 × 10^–5^ ± 2.9 × 10^–5^

Flux measured at the 24 h time point. *OS* organosilicone, *EVO* esterified vegetable oil, *FAE* fatty acid ethoxylate, and *AA* alcohol alkoxylate. *SD* standard deviation. Formulas used in the calculation of flux has been included in Additional file 5 and calculation of flux for foil, silicone and membrane mentioned in Additional file 4: Table S1

### Effects of surfactants on tracer permeability through biological membrane

**Fig. 6:**
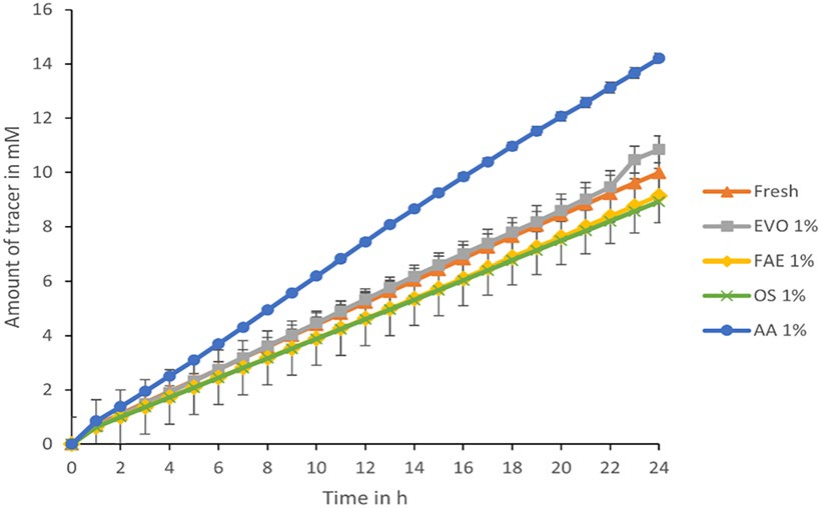
Diffusion of fluorescein sodium salt through isolated cuticular membranes in the presence of surfactants. The chart represents tracer diffusion (flux) in the presence of surfactants at 1% v/v concentration in the donor chamber over 24 h. The line marker triangle (orange colour) represents amount of tracer diffused through isolated CM without the presence of surfactants. Line marker square (grey colour) represents amount of tracer diffused in the presence of EVO. Line marker diamond (yellow colour) represents amount of tracer diffused in the presence of FAE. Line marker cross (green colour) represents tracer amount of tracer diffused in the presence of OS. Line marker round (blue colour) represents amount of tracer diffused in the presence of AA. The presence of surfactants AA, FAE, and EVO showed increased diffusion of tracer compared to OS and in the absence of surfactants (fresh). The error bar represents standard deviation.

The permeability of the tracer was evaluated in the presence of surfactants at different concentrations in the first 6 h post introduction in chamber. The permeability of the tracer in the presence or absence of surfactants showed no significant statistical differences in the flux of tracer diffused between different treatments of surfactants at 0.01% and 0.1% v/v concentrations (Table 3) and amount diffused mentioned in Additional file 6: Table S3. As the concentration of surfactants increased the flux increased moderately. Tracer permeability was the highest in the presence of esterified vegetable oil, followed by fatty acid ethoxylates. Effect of surfactants on CM at 1% v/v concentration showed stronger fluorescein penetration in the presence of AA as shown in the Fig. 6. Post diffusion experiments, visual observation of the cuticular membranes revealed intact membranes. The permeability of the tracer in the absence of surfactants was very similar to that of tracer permeability in the presence of 1% v/v of surfactants after 6 h. The tracer permeability was practically constant in the presence of alcohol alkoxylates at all three concentrations. At 24 h time point as shown in the Fig. 6, concentration of tracer was the highest in the presence of 1% v/v alcohol alkoxylate, followed by EVO, while no significant difference was found for tracer diffusion in the presence of 1% v/v concentration of the other surfactants.

**Table 3:** Tracer flux through isolated *S. lycopersicum* cuticular membranes in the presence and absence of surfactants

% v/v concentration of surfactant			
	0	0.01	0.1
Flux of tracer through isolated CM mol m^-2^s^-1^			
Donor chamber contents			
Fresh	1.6x10^-5^ ± 0.07 x 10^-5^		
EVO		3.2x10^-5^ ± 0.06 x 10^-5^	4.6x10^-5^ ± 0.9 x 10^-5^
FAE		2.9x10^-5^ ± 0.02 x 10^-5^	4.4x10^-5^ ± 0.9 x 10^-5^
AA		2.9x10^-5^ ± 0.07 x 10^-5^	2.6x10^-5^ ± 0.43 x 10^-5^
OS		3.5x10^-5^ ± 0.09 x 10^-5^	3.6x10^-5^ ± 0.85 x 10^-5^

Flux measured at the 6 h time point. *OS* organosilicone, *EVO* esterified vegetable oil, *FAE* fatty acid ethoxylate, and *AA* alcohol alkoxylate, *SD* standard deviation

## Discussion

In this study, the effect of four commercially available surfactants on tracer permeability through a biological membrane was studied using customized 3D printed diffusion chambers. The plant biological membrane chosen for diffusion studies was carefully selected after enzymatic isolation as the presence of gaps (e.g., stomata, trichome) in the cuticle can yield erroneous rate of diffusion results [36]. To avoid such errors, astomatous tomato fruit cuticular membranes were used. The enzyme isolation method used here including the acid–base washing steps resulted in mostly intact CM. All isolated CM with tears or orifices were discarded after microscopic investigation. Isolated *S. lycopersicum* fruit CM were reddish orange in colour [37]. Under the microscope apart from the thick anticlinal pegs, transparent epicuticular wax, empty epidermal cell pockets, and canals connecting adjacent epidermal cell pockets were only visible suggesting a successful cuticular membrane isolation technique. Anticlinal gaps were visible without any staining or processing steps which have been reported previously in tomato fruit cuticle using 3D imaging confocal microscopy upon staining [38] and in apple fruit cuticles using SEM [39] but has not been previously reported under light microscopy without staining.

Solvent and surfactant treatment of isolated CM resulted in mild structural changes in CM. Loss of carotenoids from the CM were visible, as the CM lost colour, when treated with chloroform and 2-propanol, along with separation of epicuticular wax resulting in reduction of membrane thickness was visible only when treated with chloroform as previously observed [40]. The thickness of the CM did not significantly reduce with the other solvent treatments. Scanning electron microscopy of surfactant treated CM showed occasional pitting when treated with FAE, AA, and EVO and surfactant flooding in the epidermal cell pockets in an otherwise empty pocket. This effect of surfactant flooding in the epidermal cell pockets has not been imaged before, and this presence of surfactant in the physiological inside of the CM is direct evidence for surfactant penetration through the CM.

Surfactant penetration through isolated CM was conducted in a 3D printed diffusion chamber custom designed and fabricated in the laboratory. The method used for printing the thermoplastics in this research is fused deposition modelling (FDM). Based on availability, known chemical stability, and finish, five thermoplastics namely- ABS (2 types), nylon, PETG and vero were tested to establish the usability of thermoplastics. FDM printed thermoplastics were systematically tested for stability against solvents and surfactants used in this research was established. For analytical applications, such as diffusion studies, it is essential to understand the interaction of these chemicals with printed materials [41] in order to eliminate chemical contamination in samples.

Chemical stability of thermoplastics against solvents as expected differed against different solvents [42, 43]; [44]. Neat concentrations of solvents the two ABS plastics, and PETG showed variable reactivities, thus suggesting an incomplete depolymerization of the plastics by the solvents. Nylon showed the highest chemical inertness [45] against the solvents as expected. On the other hand, vero magenta thermoplastic gained weight and swelled up. For this reason, vero is recommended only for modelling and prototyping purposes. Chemical instability with respect to change in weight or noticeable physical was not observed for thermoplastics treated against neat concentrations of surfactants.

The diffusion chambers were fabricated using the method fused deposition modelling (FDM) where the thermoplastics are melted, extruded through thin nozzle, layer by layer and solidified at a controlled rate. Micro-gaps occurred between parallel layers, generally not visible to the naked eye, but found using a polar dye solution. Gaps were eliminated during the production process by increasing the total number of threads (total shell number) and density as described by GIJ Salentijn, PE Oomen, M Grajewski and E Verpoorte [41]. This problem was strictly biased towards nature of the plastic used as these gaps only occurred while using ABS. PETG has a lower glass transition temperature of 80 °C and more denser hence showed a better layer adhesion, when compared to ABS [46].

Surface roughness was visible in printed ABS thermoplastics and roughness was eliminated by gently sanding the surface, followed by coating the surface with XTC-3D® resin providing a smooth finish, generally used for aesthetic purposes [47]. The resin has two components- component A is a mixture of paratertiarybutylphenol, trimethylhexamethylenediamine, 1,3-benzenemethaneeamine and para-nonylphenol and component B is made up of Oxirane,2,2’-((1-methylethylidene)-bis-(4,1-phenyleneoxymethylene)) -bis-, homopolymer. The resin after application hardened and showed softening effects only against neat concentrations of acetone and acetonitrile.

For the diffusion study, a hydrophilic tracer solute was selected for permeability experiments across various membranes. It is common practice to use radiolabelled molecule to understand diffusion kinetics through isolated plant cuticular membranes [48]. The drawbacks associated with radioactivity and the requirement of isotope facility [49] encouraged us to use fluorescent molecule (sodium salt of fluorescein). To improve accuracy of detection of the tracer, the fluorescent spectra was analysed using a Varioskan^®^ Lux multi-plate reader, which can detect femtomolar concentrations. In addition to high sensitivity, this method of analysis was much quicker, compared to using conventional methods such as HPLC or GC.

Integrity of the 3D printed thermoplastics during the permeability tests were assessed by checking tracer flux across aluminium foil and cured silicone membrane. There was no significant difference between flux established through silicone and solid aluminium sheet with both exhibiting negligible values after 24 h. There was no leakage of tracer through the printed thermoplastics, but as expected the diffusion was much more immediate and constant through the semi permeable membrane. The value of tracer flux through silicone as a membrane was important as silicone was used to bind the CM to the middle chamber and tightly shut donor-middle-receiver chamber. Hence any tracer leakage through the silicone even though negligible was estimated and was deduced from tracer flux values obtained from other experiments.

Movement of hydrophilic molecules across plant cuticular membranes have been described to occur via aqueous pores or through a random path [50–52] and the rate governed by charge on the diffusant. The intact astomatous fruit cuticular membrane of *S. lycopersicum* used in this research are known to be moderately water permeable [53] and the hydrophilic tracer used in this research readily diffused through the fruit CM. Fluorescent stains are generally used to identify stomatal infiltration of foliar sprays, but we have shown that this can be successfully used in diffusion studies as well. In addition to this, the rage of flux of the tracer estimated through our experiments corroborated to rate of flux of sucrose of similar molecular weight [54]. As expected, the flux of the tracer was higher through the dialysis membrane which has wider gaps when compared to an intact fruit cuticular membrane with aqueous pores, for example, radii of the pores are estimated to be around ~ 1.18–0.87 nm [55] in cherry skins compared to 5–10 nm in dialysis membrane.

The flux of the tracer on solvent treated cuticular membranes was higher when compared to diffusion through fresh CM. The rate of flux was nearly 8 times higher on a chloroform treated CM as the rate limiting factor the crystalline waxes were removed; a trend also noticed with lipophilic molecules diffusion through fresh and dewaxed cuticular membranes [56]. CM treated with the other solvents also showed similar increases in flux which can be attributed towards changes in the cuticular membrane [57]. The 24 h solvent treatment followed by air-drying of isolated cuticular membranes suggest changes to the rate of flux thus suggesting irreversible damage of isolated cuticular membranes. CM treated with surfactants exhibited similar increased in rate of flux of tracer. Unlike the solvent treated CM, surfactant treated CM upon air-drying still had visible deposits of surfactants (identified using SEM).

Surfactant presence in CM can be attributed to increased flux of tracer [58] by increasing the ionic permeability through lipophilic membranes. As described, in the introduction diffusion of molecules through CM is a sorption–desorption process through the various layers of CM. Commercial surfactants enhance this process by plasticizing the cuticular waxes and increasing the presence of tracer in these layers. At 0.01% and 0.1% surfactant concentrations, an increased rate of flux of tracer was noticed across the CM, when compared to flux of tracer through CM unassisted by surfactants, hence confirming the effect of surfactants on molecular permeability through isolated CM.

As the concentration of the surfactants increases in the solution the surfactants form micelles that are known to hinder the permeability of molecules. This was evident in tracer flux rate when surfactants were used at 1% v/v. It is understood that when micelles are formed, the tracer solubilizes in the micelles and less is sorbed in wax. Hence understanding the vital concentration of the tank mix significantly improves the flux of AI through cuticular membranes [17].

## Conclusion

Site directed sustainable agriculture requires accurate use of instrumentation and agrochemicals in agricultural field. Sustainable application of agrochemicals requires a thorough understanding of the permeability of chemicals through chemically resilient cuticular membranes. As a proof of concept, the molecular permeability of a tracer was studied in the presence of commercial surfactants through isolated plant cuticular membranes in custom printed 3D chambers in this research. A range of thermoplastics were profiled, and suitable materials were identified for diffusion chamber fabrication. Diffusion of fluorescent ionic solute was carried out through enzymatically isolated slightly hydrophilic cuticle and the tracer flux was determined through UV spectroscopy. Solvent and surfactant treated cuticles readily allowed tracer permeability showing up to 8 times increase in flux. The effect of lipophilic surfactants at commercially recommended concentrations on tracer permeability doubled the flux. The current research demonstrates the flexibility around designing and manufacturing a customized diffusion chamber for analysis. We are confident that information obtained from this research can be used as a platform to develop an in depth understanding of functions of individual components of surfactants in the future.

## Supplementary Information

**Additional file 1: Figure S1**. Chamber design. 2D CAD drawings of different parts of the diffusion chamber.

**Additional file 2: Figure S2.1**: Screenshot of the initial drawing steps. **Figure S2.2**: Screenshot of initial sketching phase of a 2D drawing on one of the planes. **Figure S2.3**: Screenshot showing a polygon with the respective parameters. **Figure S2.4**: Screenshot showing the extrusion of the polygon to form a solid 3D object. **Figure S2.5**: Screenshot showing solid block and reverse extrusion of a circle to form hollow interior chamber. **Figure S2.6**: Screenshot showing hollow chamber and selection steps for drawing the sample port. **Figure S2.7**: Screenshot showing the sampling port extrusion and translucent solid block showing the interior elements.

**Additional file 3: Figure S3**. Effect of solvents and surfactants on printed thermoplastics.

**Additional file 4: Table S1. **Calculation of flux of the tracer through different materials.

**Additional file 5: Table S2. **Amount of fluorescein sodium salt diffused after 6 h.

**Additional file 6: Table S3. **Amount of fluorescein sodium salt diffused after 6 h.
