# Supplementary figures and images for: Analysis of plant cuticles and their interactions with agrochemical surfactants using a 3D printed diffusion chamber
Source: Plant Methods. 2023 Apr 1;19:37. doi: 10.1186/s13007-023-00999-y (PMC10067233; doi:10.1186/s13007-023-00999-y)

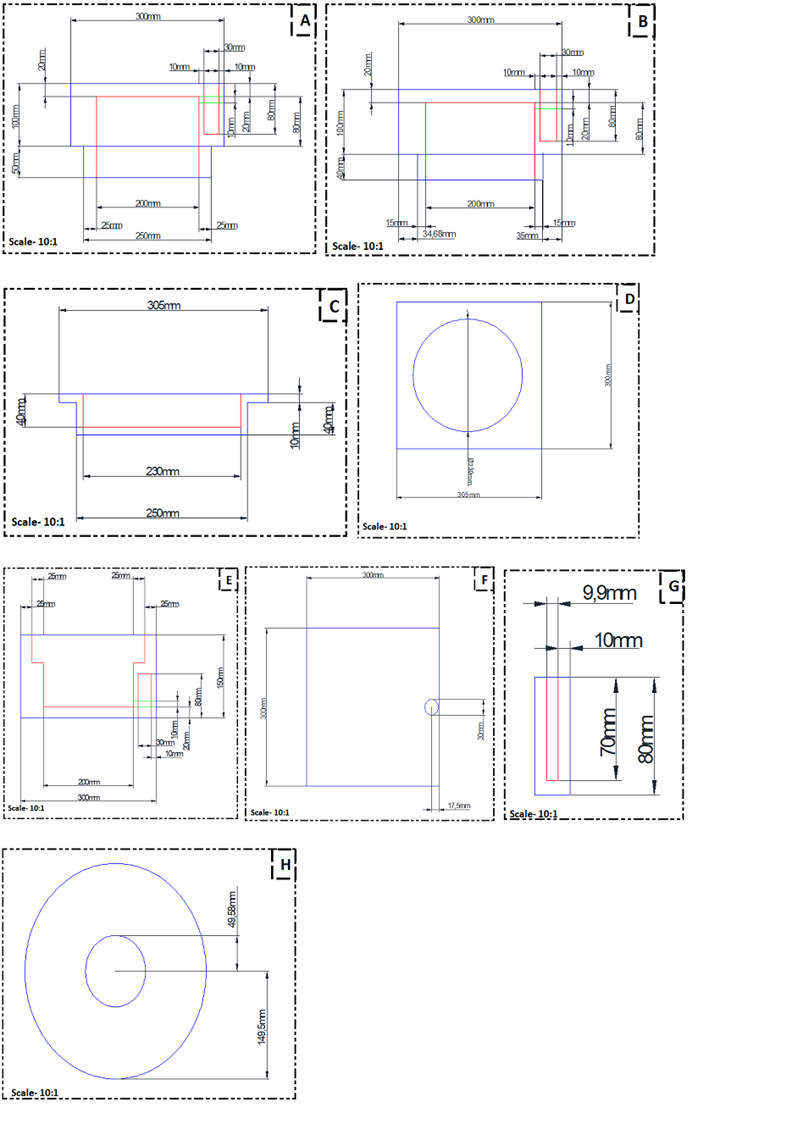

Supplement: Supplementary file 1 — Additional file 1: Figure S1. Chamber design. 2D CAD drawings of different parts of the diffusion chamber. [file 13007_2023_999_MOESM1_ESM.tif]

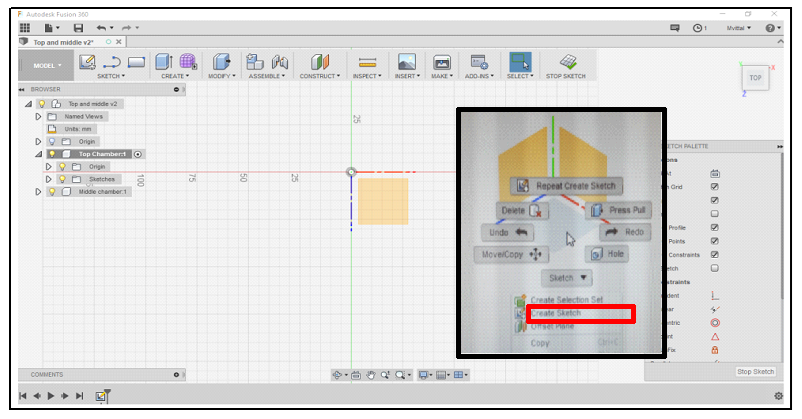

Supplement: Supplementary file 2 — Additional file 2: Figure S2.1: Screenshot of the initial drawing steps. Figure S2.2: Screenshot of initial sketching phase of a 2D drawing on one of the planes. Figure S2.3: Screenshot showing a polygon with the respective parameters. Figure S2.4: Screenshot showing the extrusion of the polygon to form a solid 3D object. Figure S2.5: Screenshot showing solid block and reverse extrusion of a circle to form hollow interior chamber. Figure S2.6: Screenshot showing hollow chamber and selection steps for drawing the sample port. Figure S2.7: Screenshot showing the sampling port extrusion and translucent solid block showing the interior elements. [file 13007_2023_999_MOESM2_ESM.zip › Figure S2-2 Screenshot of initial sketching phase of a 2D drawing on one of the planes.tif]

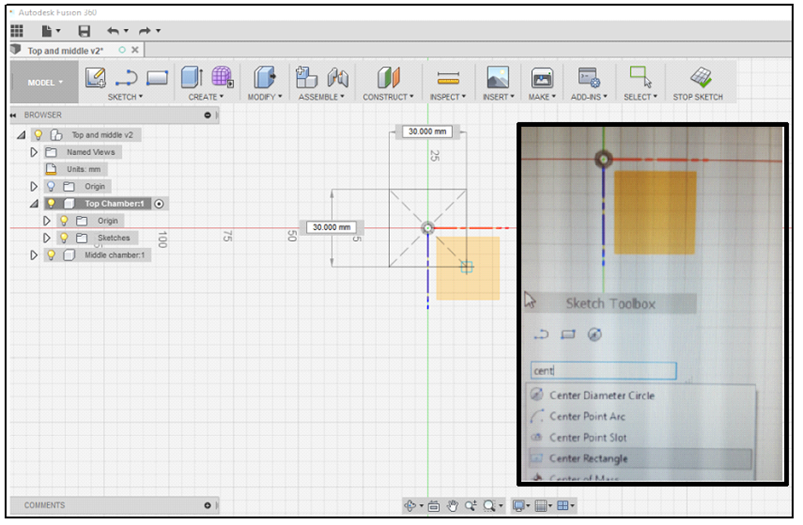

Supplement: Supplementary file 2 — Additional file 2: Figure S2.1: Screenshot of the initial drawing steps. Figure S2.2: Screenshot of initial sketching phase of a 2D drawing on one of the planes. Figure S2.3: Screenshot showing a polygon with the respective parameters. Figure S2.4: Screenshot showing the extrusion of the polygon to form a solid 3D object. Figure S2.5: Screenshot showing solid block and reverse extrusion of a circle to form hollow interior chamber. Figure S2.6: Screenshot showing hollow chamber and selection steps for drawing the sample port. Figure S2.7: Screenshot showing the sampling port extrusion and translucent solid block showing the interior elements. [file 13007_2023_999_MOESM2_ESM.zip › Figure S2-3 Screenshot showing a polygon with the respective parameters.tif]

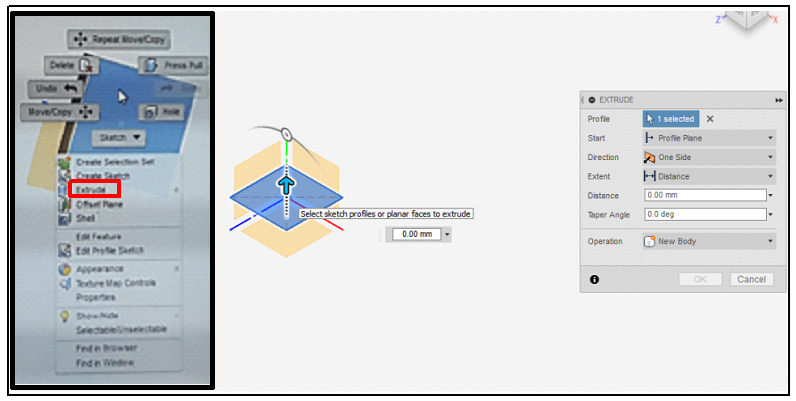

Supplement: Supplementary file 2 — Additional file 2: Figure S2.1: Screenshot of the initial drawing steps. Figure S2.2: Screenshot of initial sketching phase of a 2D drawing on one of the planes. Figure S2.3: Screenshot showing a polygon with the respective parameters. Figure S2.4: Screenshot showing the extrusion of the polygon to form a solid 3D object. Figure S2.5: Screenshot showing solid block and reverse extrusion of a circle to form hollow interior chamber. Figure S2.6: Screenshot showing hollow chamber and selection steps for drawing the sample port. Figure S2.7: Screenshot showing the sampling port extrusion and translucent solid block showing the interior elements. [file 13007_2023_999_MOESM2_ESM.zip › Figure S2-4 Screenshot showing the extrusion of the polygon to form a solid 3D object.tif]

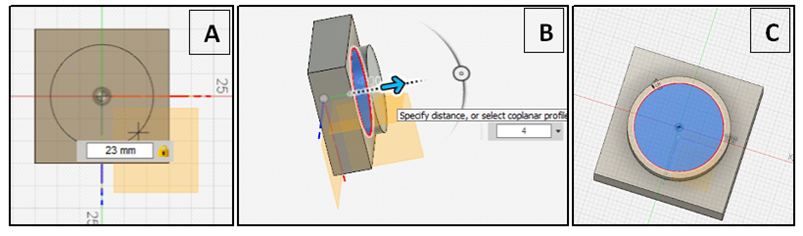

Supplement: Supplementary file 2 — Additional file 2: Figure S2.1: Screenshot of the initial drawing steps. Figure S2.2: Screenshot of initial sketching phase of a 2D drawing on one of the planes. Figure S2.3: Screenshot showing a polygon with the respective parameters. Figure S2.4: Screenshot showing the extrusion of the polygon to form a solid 3D object. Figure S2.5: Screenshot showing solid block and reverse extrusion of a circle to form hollow interior chamber. Figure S2.6: Screenshot showing hollow chamber and selection steps for drawing the sample port. Figure S2.7: Screenshot showing the sampling port extrusion and translucent solid block showing the interior elements. [file 13007_2023_999_MOESM2_ESM.zip › Figure S2-5 Screenshot showing solid block and reverse extrusion of a circle to form hollow interior chamber.tif]

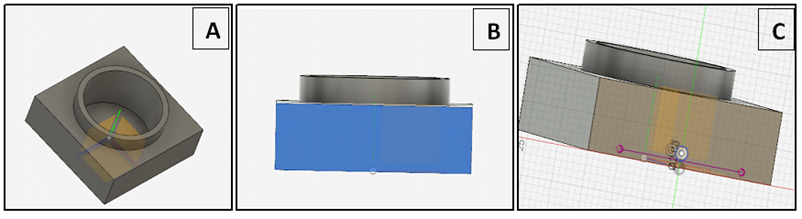

Supplement: Supplementary file 2 — Additional file 2: Figure S2.1: Screenshot of the initial drawing steps. Figure S2.2: Screenshot of initial sketching phase of a 2D drawing on one of the planes. Figure S2.3: Screenshot showing a polygon with the respective parameters. Figure S2.4: Screenshot showing the extrusion of the polygon to form a solid 3D object. Figure S2.5: Screenshot showing solid block and reverse extrusion of a circle to form hollow interior chamber. Figure S2.6: Screenshot showing hollow chamber and selection steps for drawing the sample port. Figure S2.7: Screenshot showing the sampling port extrusion and translucent solid block showing the interior elements. [file 13007_2023_999_MOESM2_ESM.zip › Figure S2-6 Screenshot showing hollow chamber and selection steps for drawin the sample port.tif]

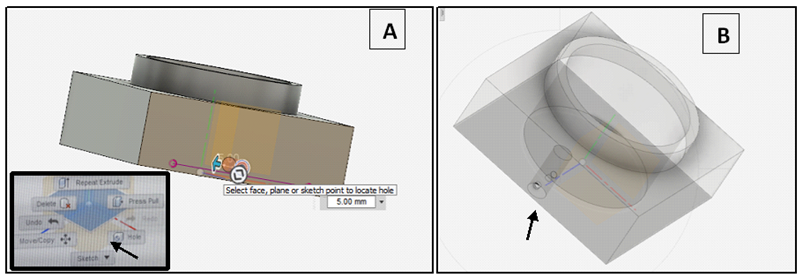

Supplement: Supplementary file 2 — Additional file 2: Figure S2.1: Screenshot of the initial drawing steps. Figure S2.2: Screenshot of initial sketching phase of a 2D drawing on one of the planes. Figure S2.3: Screenshot showing a polygon with the respective parameters. Figure S2.4: Screenshot showing the extrusion of the polygon to form a solid 3D object. Figure S2.5: Screenshot showing solid block and reverse extrusion of a circle to form hollow interior chamber. Figure S2.6: Screenshot showing hollow chamber and selection steps for drawing the sample port. Figure S2.7: Screenshot showing the sampling port extrusion and translucent solid block showing the interior elements. [file 13007_2023_999_MOESM2_ESM.zip › Figure S2-7 Screenshot showing the sampling port extrusion and translucent solid block showing the interior elements.tif]

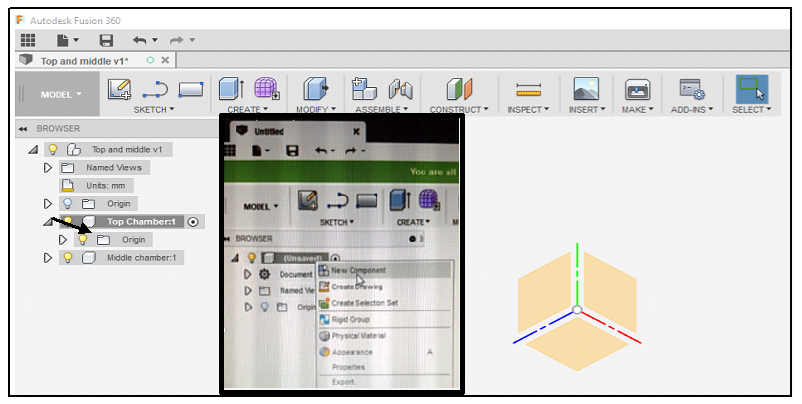

Supplement: Supplementary file 2 — Additional file 2: Figure S2.1: Screenshot of the initial drawing steps. Figure S2.2: Screenshot of initial sketching phase of a 2D drawing on one of the planes. Figure S2.3: Screenshot showing a polygon with the respective parameters. Figure S2.4: Screenshot showing the extrusion of the polygon to form a solid 3D object. Figure S2.5: Screenshot showing solid block and reverse extrusion of a circle to form hollow interior chamber. Figure S2.6: Screenshot showing hollow chamber and selection steps for drawing the sample port. Figure S2.7: Screenshot showing the sampling port extrusion and translucent solid block showing the interior elements. [file 13007_2023_999_MOESM2_ESM.zip › 2-Figure S2-1 Screenshot of the initial drawing steps.tif]

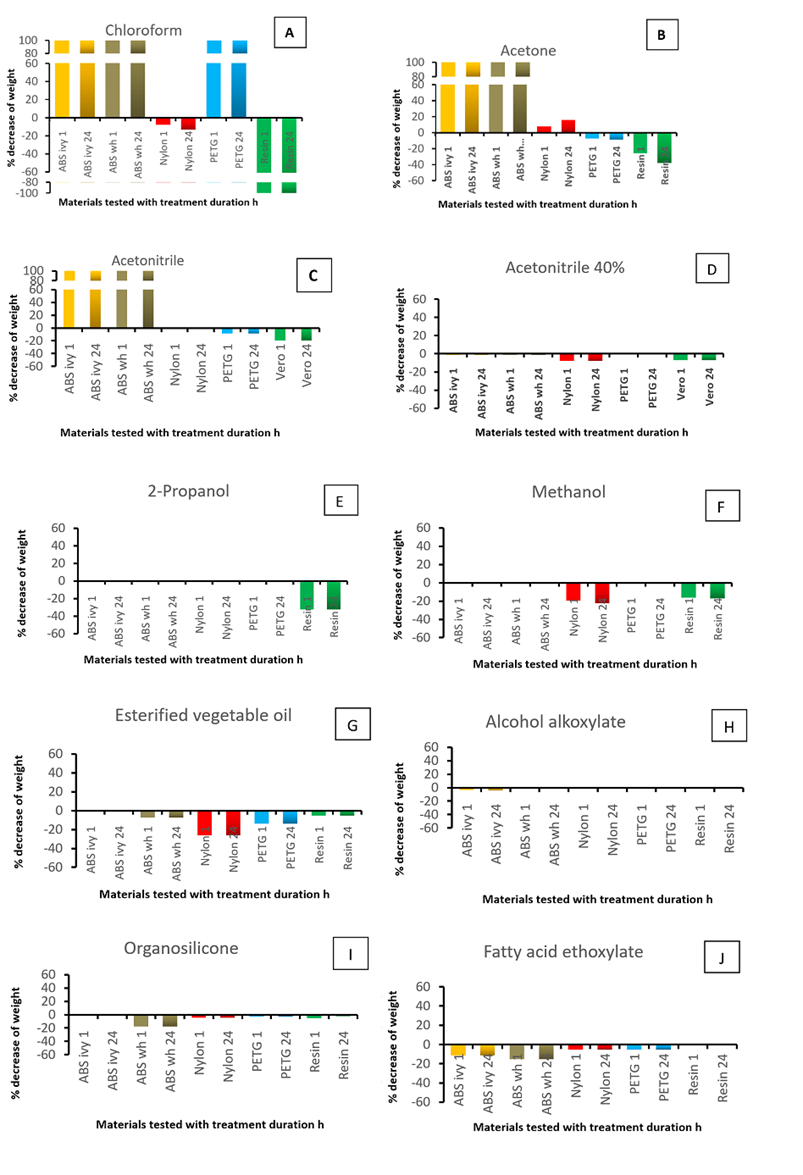

Supplement: Supplementary file 3 — Additional file 3: Figure S3. Effect of solvents and surfactants on printed thermoplastics. [file 13007_2023_999_MOESM3_ESM.tif]
