# Supplementary material for: Analysis of plant cuticles and their interactions with agrochemical surfactants using a 3D printed diffusion chamber
Source: Plant Methods. 2023 Apr 1;19:37. doi: 10.1186/s13007-023-00999-y (PMC10067233; doi:10.1186/s13007-023-00999-y)
Supplement: Supplementary file 4 — Additional file 4: Table S1. Calculation of flux of the tracer through different materials. [file 13007_2023_999_MOESM4_ESM.docx]

**Table S1. Calculation of flux of the tracer through different materials.**

| **Parameters** | **Foil** | **Silicone** | **Membrane** |
| --- | --- | --- | --- |
| Thickness **(µm)** | 21.22 ± 5.6 | 463.40 ± 6.9 | 20 |
| Estimated holdup time t_e_ **(h)** | 20 | 9.6 | 2.7 x 10^-4^ |
| Amount of tracer diffused (M) in **(mol/h)** | 1.9 x 10^-10^ | 1.9 x 10^-9^ | 2.44x 10^-7^ |
| Area A **(m^2^)** | 0.0079 | 0.0079 m^2^ | 0.0079 m^2^ |
| Flux J **(mol m^-2^ s^-1^)** | 6.8 x 10^-11^ | 6.57 x 10^-11^ | 8.56 x 10^-5^ |

Table legend- Estimated hold up time t_e_ = 20h. The amount of tracer diffusing obtained from the slope of the y intercept. M = 4.68 x 10^-8^ mol/24 h; M= 1.95 x 10^-9^ mol/ h; M/t = 5.41 x 10^-13^ mol s^-1^; Area of the orifice exposing the foil A= 0.0079 m^2^; and Calculation of flux J= (M/t) ꓫ (1/A).
