# Supplementary material for: Analysis of plant cuticles and their interactions with agrochemical surfactants using a 3D printed diffusion chamber
Source: Plant Methods. 2023 Apr 1;19:37. doi: 10.1186/s13007-023-00999-y (PMC10067233; doi:10.1186/s13007-023-00999-y)
Supplement: Supplementary file 5 — Additional file 5: Table S2. Amount of fluorescein sodium salt diffused after 6 h. [file 13007_2023_999_MOESM5_ESM.docx]

**Table S2. Amount of fluorescein sodium salt diffused after 6 h**

| **Material** | **Treatment** | **Amount of tracer mM± SD** |
| --- | --- | --- |
| ***Biological membrane*** |  |  |
| CM | Fresh | 2.7 ± 0.6 |
|  | ***Solvents*** |  |
| CM | Ethanol | 6.5 ± 0.5 |
| CM | Chloroform | 7.8 ± 0.4 |
| CM | Acetonitrile | 7.8 ± 0.4 |
| CM | Acetone | 9.4 ± 0.5 |
| CM | 2- Propanol | 9.3 ± 0.7 |
|  | ***Surfactants*** |  |
| CM | OS | 3.4 ± 1.04 |
| CM | FAE | 7.1 ± 0.3 |
| CM | AA | 10.1 ± 0.8 |
| CM | EVO | 12.3 ± 0.6 |

Table legend- Estimated amount of tracer diffused after 6h of diffusion though fresh and treated tomato fruit cuticular membranes with standard deviations. For non-biological material the amount of Fluorescein sodium salt diffused was as follows Foil- 0.2 μM after 24 h, Silicone 1.8μM after 24 h, Semi permeable membrane 7.8 mM after 6 h.
