# Supplementary material for: Analysis of plant cuticles and their interactions with agrochemical surfactants using a 3D printed diffusion chamber
Source: Plant Methods. 2023 Apr 1;19:37. doi: 10.1186/s13007-023-00999-y (PMC10067233; doi:10.1186/s13007-023-00999-y)
Supplement: Supplementary file 6 — Additional file 6: Table S3. Amount of fluorescein sodium salt diffused after 6 h. [file 13007_2023_999_MOESM6_ESM.docx]

**Table S3. Amount of fluorescein sodium salt diffused after 6 h**

| **% Concentration of surfactant (v/v)** | **Amount of fluorescein diffused in mM ± SD** | | | |
| --- | --- | --- | --- | --- |
| 0 | 2.7 ± 0.6 | | | |
|  | **Surfactant type** | | | |
|  | **EVO** | **FAE** | **AA** | **OS** |
| 0.01 | 4.5 ± 0.9 | 4.1± 0.2 | 4.1 ± 0.9 | 5 ± 1.3 |
| 0.1 | 6.5 ± 1.4 | 6.32 ± 1.3 | 3.76 ± 0.6 | 5.08 ± 1.2 |
| 1 | 2.76 ± 0.1 | 2.46 ± 0.01 | 3.7 ± 0.04 | 2.44 ± 0.01 |

Table legend- Table represents the amount of AI (fluorescein sodium salt) diffused after 6 h in the absence and presence of various surfactants and at different concentrations, through tomato fruit cuticular membrane (± Standard Deviation). 0% v/v surfactant concentration represents average amount of AI concentration found after 6h of diffusion analysis. Surfactants abbreviations- EVO- esterified vegetable oil, FAE- Fatty acid ethoxylate, AA- alkyl alkoxylate, and OS- organosilicone surfactant.
